# The application of omics technologies for understanding tropical plants-based bioactive compounds in ruminants: a review

**DOI:** 10.1186/s40104-024-01017-4

**Published:** 2024-05-01

**Authors:** Metha Wanapat, Gamonmas Dagaew, Sukruthai Sommai, Maharach Matra, Chaichana Suriyapha, Rittikeard Prachumchai, Uswatun Muslykhah, Srisan Phupaboon

**Affiliations:** 1 Tropical Feed Resources Research and Development Center (TROFREC), Department of Animal Science, Faculty of Agriculture, Khon Kaen University, Khon Kaen, 40002 Thailand; 2 Department of Animal Science, Faculty of Agricultural Technology, University of Technology Thanyaburi, Rajamangala Pathum Thani, 12130 Thailand

**Keywords:** Animal nutrition, Animal production, Cutting-edge, Molecular markers, Ruminants

## Abstract

Finding out how diet impacts health and metabolism while concentrating on the functional qualities and bioactive components of food is the crucial scientific objective of nutritional research. The complex relationship between metabolism and nutrition could be investigated with cutting-edge "omics" and bioinformatics techniques. This review paper provides an overview of the use of omics technologies in nutritional research, with a particular emphasis on the new applications of transcriptomics, proteomics, metabolomics, and genomes in functional and biological activity research on ruminant livestock and products in the tropical regions. A wealth of knowledge has been uncovered regarding the regulation and use of numerous physiological and pathological processes by gene, mRNA, protein, and metabolite expressions under various physiological situations and guidelines. In particular, the components of meat and milk were assessed using omics research utilizing the various methods of transcriptomics, proteomics, metabolomics, and genomes. The goal of this review is to use omics technologies—which have been steadily gaining popularity as technological tools—to develop new nutritional, genetic, and leadership strategies to improve animal products and their quality control. We also present an overview of the new applications of omics technologies in cattle production and employ nutriomics and foodomics technologies to investigate the microbes in the rumen ecology. Thus, the application of state-of-the-art omics technology may aid in our understanding of how species and/or breeds adapt, and the sustainability of tropical animal production, in the long run, is becoming increasingly important as a means of mitigating the consequences of climate change.

## Introduction

Currently, animal nutrition researchers have an idea of combining knowledge of nutritive values with cutting-edge technologies, especially “omics technologies”, to be used as a technological tool to evaluate the general consumption rates, diversity of the microbial population, the effects of feed or feed additives on animal health and the quality control of animal products like milk and meat. Significant technical advancements over time have aided in the advancement of dairy-related research on data-driven omics techniques like nutriomics and foodomics [1]. Recent research on intricate and interconnected variables affecting meat and milk safety is increasingly centered on data-driven omics techniques using proteomics [2].

The omics developments in defining the biochemical and biological effects, particularly bioactive compounds, on feed nutrition safety are highlighted in this review. Additionally, the effects of meat and milk nutritional qualities on human health are highlighted. According to the descriptions of Munekata et al. [3], bioactive substances play a significant role in the secondary metabolism of plants, particularly in tropical plants that are higher in bioactive compounds influenced by climate change. These compounds have a variety of biological impacts on animal health. As a result, mounting data suggests that ruminants consuming these bioactive compounds cannot have adverse effects while directly inhibiting methanogen-archive growth in the rumen [4, 5]. Several substances from tropical plants and/or dairy foods, including polyphenols, glycosylates/isothiocyanates, anthocyanins, carotenoids and other terpenoids, alkaloids, dietary fiber, poly/mono-peptides, vitamins, and α/β-lactoferrin, can be categorized as being bioactive compounds [5, 6]. High-throughput technologies were used to look at genetics, proteins, and metabolites in these results. This was done to help fill in methodological gaps in biological or bioactive research and give researchers the tools they need to look into the quality of meat and milk [2, 7].

Therefore, our commitment in presenting this review article is to bring together modern technology or cutting-edge model. To investigate the characterization of bioactive compounds using genomic, transcriptomic, proteomic, and metabolomic approaches. By fermenting rumen microorganisms and utilizing omics technology, statistical and mega-database insights are employed to link formulas for predicting the meat/milk quality and biologically active substances in meat and milk.

## Use of bioactive compounds in ruminant feed supplement

The livestock production industry is under increasing pressure to improve efficiency to satisfy the need of feed a growing population from a base of limited resources [8]. To achieve optimal anaerobic fermentation in the rumen, it is necessary to maintain a consistent provision of substrate in terms of both quantity and frequency. Additionally, it is crucial to create and sustain a favorable environment that promotes microbial growth, which includes controlling factors such as temperature, pH, and substrate mixing. Furthermore, the continuous elimination of undesirable substances, such as bacterial toxins and hydrogen, is essential for the overall efficiency of the fermentation process. Nutritional manipulation through feed formulation and feeding management, particularly utilizing plant extracts or plants containing secondary compounds (condensed tannins and saponins) and plant oils, produces volatile fatty acids (VFAs) and microbial proteins while also reducing methane production in the rumen [9]. Whether or not these conditions are present, the rumen can function, but it may not be maintaining a healthy, functional rumen or operating at its maximum anaerobic efficiency [10]. This is where rumen modifiers, represented by feed additives, come into play. Because they are unrelated to critical plant functions such as photosynthesis, respiration, growth, and development, they are referred to as plant secondary metabolites or bioactive compounds [11].

In general, bioactive compounds (for example, polyphenolics), terpenoids (for example, terpenes), and alkaloids are categorized as phytochemicals of nutritional and pharmacological significance, such as those to treat (photo-chemotherapeutic) or prevent (phyto-chemoprophylaxis) animal diseases [11]. Although not all phytochemicals have been found to have positive effects on ruminants, the ones that do are often classified as polyphenolics (including condensed tannins), terpenoids (such as saponins), vitamins, and essential oils. The task of documenting and studying the impacts on economically valuable animal species is a highly complex endeavor, mostly attributable to the extensive diversity in phytochemical and biological characteristics [10]. The influence of tannin supplementation on methane (CH_4_) mitigation is that both condensed tannins and hydrolysable tannins possess characteristics that inhibit methanogenesis. Tannins present in methanogens exhibit two distinct mechanisms of action: a direct influence on methanogens residing in the rumen as well as an indirect effect on hydrogen production resulting from reduced feed degradation [10–12]. In addition, Wanapat et al. [12] have provided evidence suggesting that certain condensed tannins possess the ability to mitigate methane production, alleviate bloating, and enhance the absorption of amino acids within the intestines. Saponins have a crucial role in modulating rumen fermentation, principally through the mitigation of protein degradation and the reduction of concentrations of rumen urea and ammonia. Consequently, this leads to an augmented flow of amino acids into the small intestine. The possible impacts of saponins are associated with nitrogen metabolism, principally attributed to their fatal effects on protozoa, which play a crucial role in rumen proteolytic activity [13]. According to Jayanegara et al. [14], the effect of saponins on methanogens does not necessarily align with their effect on protozoa. However, saponins have been seen to reduce the population of some methanogens that are associated with protozoa. The correlation between the antiprotozoal impact of saponins and the interaction between the sterol moiety and saponins present in the protozoan membrane has been observed [13].

**Fig. 1:**
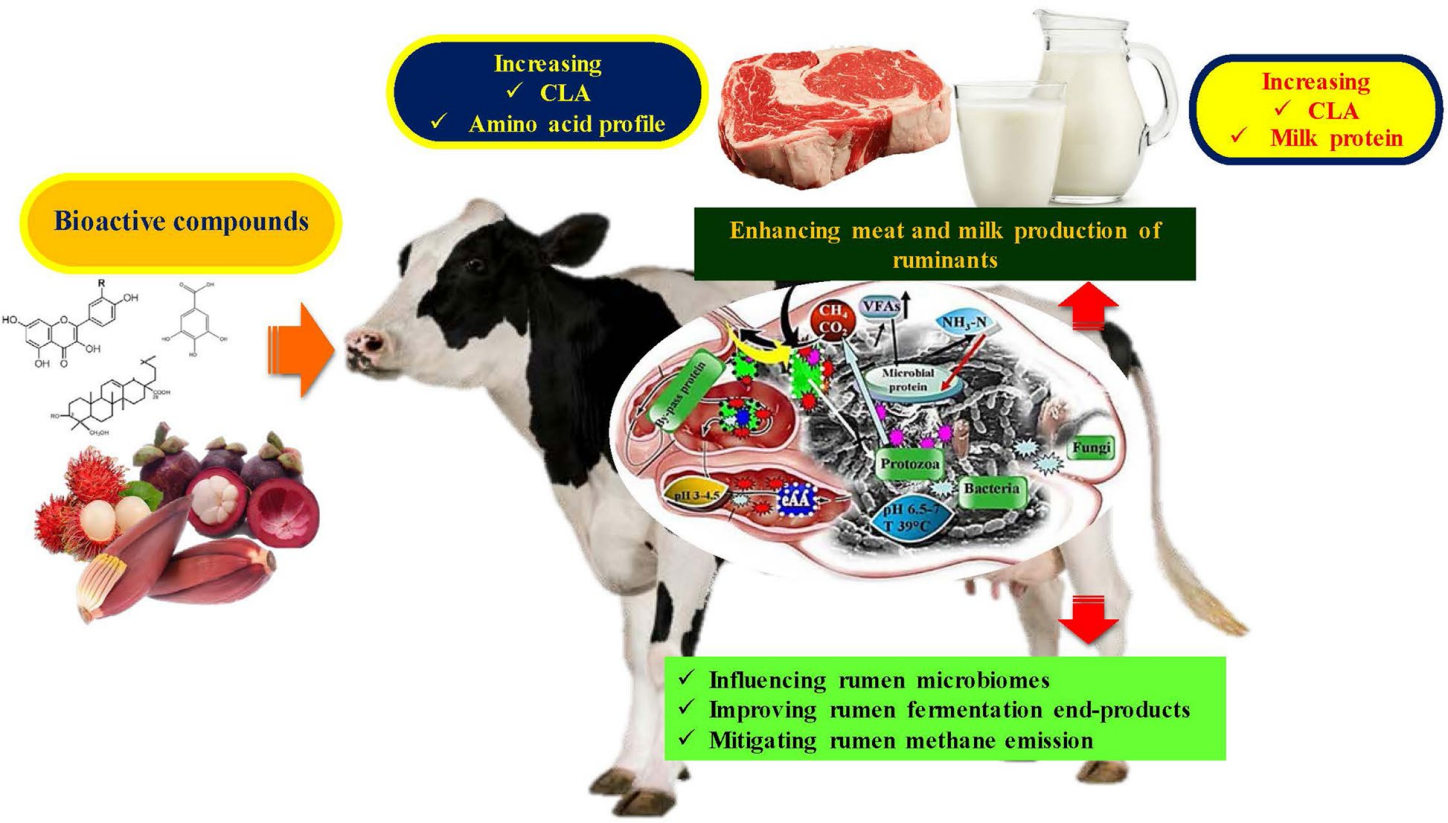
Mode of action of bioactive compounds in ruminants modified from Wanapat [22]

Ampapon et al. [15] demonstrated that the addition of phytonutrients (PNT) such as polyphenols and flavonoids to a diet has the potential to enhance fiber digestibility, promote the growth of proteolytic and cellulolytic bacteria, and induce changes in rumen VFA composition. Specifically, there is evidence of enhanced propionate concentration, VFA production and reduced methane generation as a result of PNT supplementation. The study conducted by Chanjula et al. [16] provided evidence supporting the presence of dried *Mitragyna speciosa*, a herbaceous plant predominantly distributed in the southern parts of Thailand. The presence of phytonutrient components in this particular plant may potentially confer benefits to ruminant animals. The impacts of tropical plant phytonutrients on rumen fermentation and methane production have been observed, as noted by Matra et al. [17]. The composition of dragon fruit peel powder (DFPP) consists of condensed tannins (CT) and saponins (SP). The inclusion of DFPP and a non-protein nitrogen source has the potential to boost rumen fermentation, improve the degradability of nutrients, and establish DFPP as a viable dietary rumen enhancer. Matra and Wanapat [18] conducted a study on the effects of phytonutrient pellet supplementation on rumen fermentation efficiency and milk production in lactating Holstein–Friesian crossbred cows. The utilization of DFPP as a supplement resulted in enhanced microbial protein synthesis and elevated milk fat content. However, the supplementation of DFPP only contributed to an increase in milk output and 3.5% fat-adjusted milk yield. In a previous study, glucose was identified as the predominant lipid precursor in intramuscular adipose tissue, while acetate was found to contribute the highest proportion to lipogenesis in subcutaneous adipose tissue [19]. Fatty acids constitute the primary constituents of lipids, exerting a discernible influence on the meat’s overall quality. The quality of fatty acids is significantly influenced by their concentration, thereby determining their saturation level. The composition of fatty acids in biological tissues can be affected by diet, as evidenced by a study where goats that were fed pasture exhibited a higher proportion of unsaturated intermuscular fat compared to those that were given grain [20]. Accordance with Álvarez-Rodríguez et al. [21] reported that significant dietary incorporation of proanthocyanins, phenolic compounds, and terpenes has been observed to decrease lipid oxidation in muscle tissue, potentially enhancing the longevity of lamb meat. This effect is likely due to a synergistic interaction with dietary vitamin E. However, the appropriate dietary inclusion levels are contingent upon the concentration of polyphenols and antioxidant capacity found in the feedstuffs. The inclusion of bioactive compounds in ruminant diets can impact both the rumen microbial environment and the performance of the ruminant [15]. In general, bioactive compounds have the ability to alter fermentation characteristics. They can also operate as antimicrobial agents, reducing the presence of pathogens and methane-producing bacteria. Additionally, bioactive compounds can enhance the antioxidant content, thereby reducing lipid oxidation and improving the quality of milk and meat production by mode of action in rumen fermentation, as shown in Fig. 1 [22].

## Use of omics technology to analyze bioactive compounds in ruminant products

**Fig. 2:**
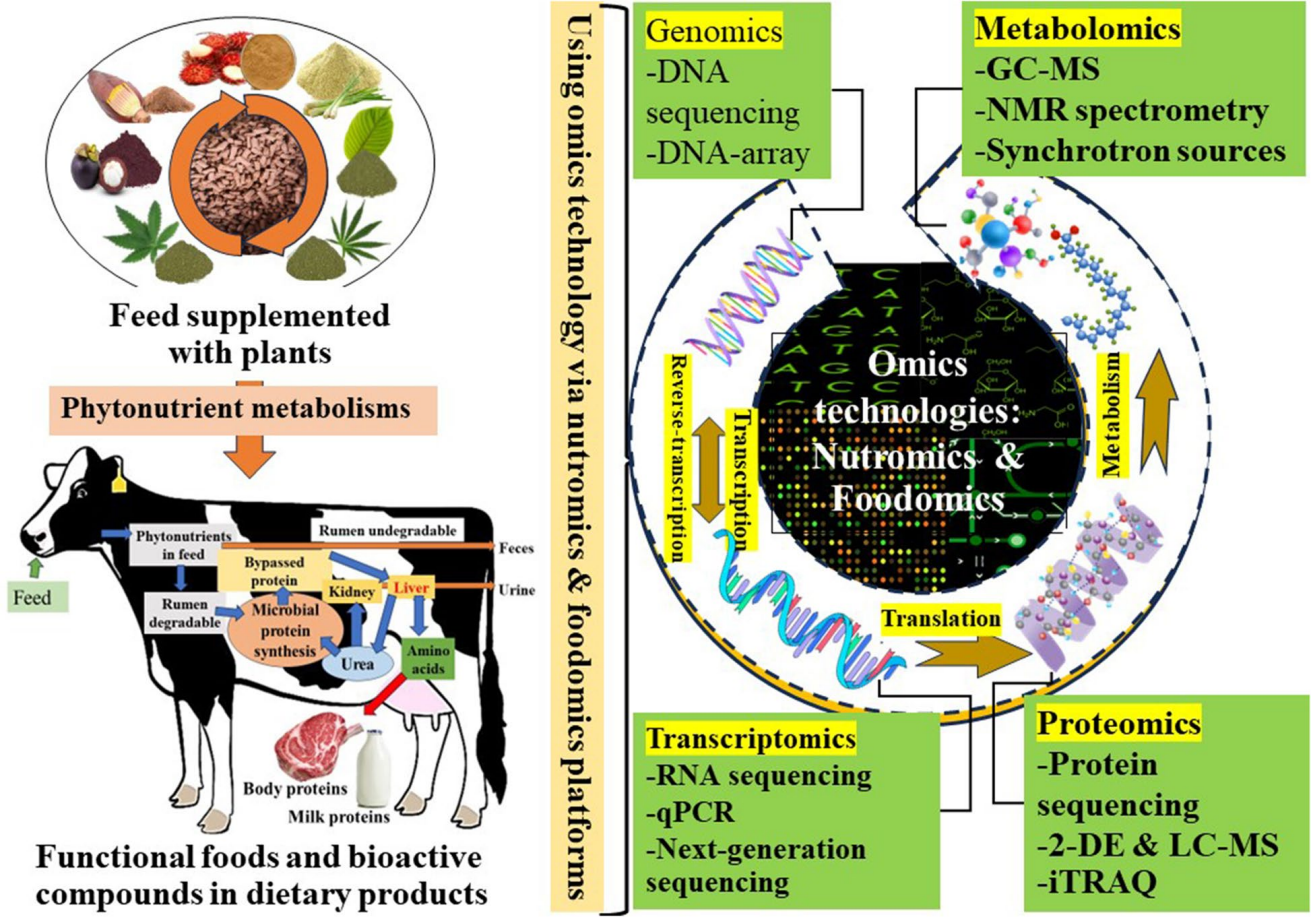
Relationship between nutromics and foodomics technologies in nutrition research

Omics technology refers to the molecular application of the nutrition-to-gene relationship, incorporating more extensive data from molecular genetics, animal nutrition, and veterinary sciences. This technology was developed through the genomics, transcriptomics, proteomics, and metabolomics in biological research. Data sources of biological information that have been gathered regarding the changes in gene, mRNA, and protein expressions as well as metabolites in different physiological conditions and regulations. This has dramatically advanced our understanding of the regulation of many physiological and pathophysiological processes [23, 24]. System biology technology, for example, has been created to integrate all data at the mRNA, protein, and metabolite levels, generating route information and enabling the measurement of tiny perturbations of pathways caused by nutrients [25]. These omics technologies' ultimate objective is to find the molecular signatures of dietary nutrients and non-nutrients that contribute to a particular phenotype and to provide dietary advice for individualized health maintenance and illness prevention. Therefore, this paper introduces omics technologies in nutrition/food research and focuses on contemporary genomics, transcriptomics, proteomics, and metabolomics applications. For example, many special omics technological tools: DNA or RNA-sequencing such as next generation sequencing (NGS), 2-dimensional gel electrophoresis (2-DE) combined with mass spectrophotometer (MS), and gas chromatography-mass spectrometry (GC–MS) as shown in Fig. 2.

### Genomics and transcriptomics

The term "genomics" refers to the methodology used to map, sequence, and analyze all genes (such as DNA) found in a particular species’ genome. The relationship between an animal feed and genome is known as nutrigenomics [26]. Understanding the physiological, biochemical, and metabolic processes when examining the responses of organisms to dietary components is required in order to examine the importance of food and diet formulation [26, 27]. Consequently, studying an organism’s entire transcriptome, or RNA products, is known as transcriptomics. The tool was widely used in transcriptomics is qRT-PCR and/or microarray, which enables the simultaneous measurement of the expression levels of up to 50,000 transcripts. In order to determine the cellular reactions to food components and their molecular targets, DNA or RNA microarray technology has been used in some cases in cell culture systems or with laboratory animals [1]. In general, DNA microarray is a complex method that measures the level of gene or mRNA expression one time. The hybridization of two pieces of conventional DNA, which are distinguished by covalent or hydrogen bonds, is the basis for DNA arrays. Many fluorescent probes are used to measure gene expression, and these probes are divided into two classes based on how an array works: single labeling and multiple labeling [28]. A summary of the previous tools used in nutrigenomic application has been applied in lactation research, especially with dairy cows (see Table 1 for details).

**Table 1:** List of genomic application in milk products and milk composition based on dairy cow research

**Methodology**	**Sample source**	**Target genes**	**Ref**
Milk production			
Molecular approaches	Cattle whole genome	The ras-related protein rap-1A on bovine chromosome 3, insulin-like growth factor 2	[29, 30]
Milk composition			
Bayesian stochastic search variable selection model	Milk	DGAT1, SCD-1 A293 V polymorphisms, SREBP-1	[31–33]
Animal model in ASReml or capillary zone electrophoresis	Milk	β-CN genotype and the κ-CN genotype, variants of the β-LG genotype B and the β-κ-CN haplotype A2B	[34, 35]

*DGAT1* Diacylglycerol-acyl transferase 1, *SCD* Stearoyl CoA desaturase, *SREBP-1* Sterol regulatory element-binding transcription factor 1, *CN* Casein, *LG* Lactoglobulin

### Proteomics

The study of all expressed proteins, together with their interactions with one another in a particular cell or tissue, as well as all protein isoforms and changes, is known as proteomics. With the help of this technique, it is possible to characterize the diversity of protein structure and the connection between this diversity and underlying biological functions, which is also employed in research on possible diseases and the diet [36]. In actuality, proteomics is a more direct method than genomics and transcriptomics for studying biological processes. Milk components, protein profiles, milk features, the effects of nutrition and lactation stages on milk synthesis, have all been studied using proteomics methodology (Table 2).

**Table 2:** List of proteomic applications on synthesis of milk products at different stages of lactation

**Methodology**	**Protein type**	**Species and stage of lactation**	**Target genes**	**Ref**
Milk components				
SDS-PAGE, MS	MFGM	Mid-lactation	Cell signaling and membrane/ Protein Trafficking	[37]
Shotgun proteomics	MFGM	D 7 after calving compared with colostrum	Lipid transportation, synthesis, and secretion	[38]
2D, MS	Whey proteome, milk protein	Early lactation, colostrum, caprine, bovine, equine, buffalo	Immunoglobulins and caseins β-lactoglobumin, β2-microglobulin, Vitamin D-binding protein, zinc-α-2-glycoprotein, and immunoglobulin G2 chain C	[39, 40]
Nutrition and lactation stages				
2D, MS	Milk protein	Lys and Met (Cow, Mid-lactation stage to compare quality in fresh milk with colostrum)	A series of biological processes such as transcription, translation, protein synthesis, cell division and differentiation and even the cell cycle	[41]

*MS* Mass spectrometer, *SDS-PAGE* Sodium dodecyl sulfate polyacrylamide gel electrophoresis, *2D* 2-D electrophoresis, *MFGM* Milk fat globule membrane, *Lys* Lysine, *Met* Methionine

### Metabolomics

The full examination of the entire metabolome is known as "metabolomics" (all metabolites synthesized by an organism). The study of metabolomics uses biochemical analytical methods to characterize the changes of small molecules (1.5 kDa) in metabolites to determine the physiological states of the animals [42]. Metabolic concentrations and their kinetic variations in cells, tissues, and organs reflect the actual end points of physiological regulatory processes, whereas gene expression or protein concentration may simply represent the potential for physiological change. Despite its early development in the field of nutrition research, nutrimetabolomics has already provided intriguing insights to comprehend the metabolic reactions of people or animals to dietary treatments [1]. Several areas of research have proven that metabolite biomarkers and milk or lactation phases are related (summarized in Table 3).

**Table 3:** List of metabolomic application in milk products and quality of milk based on dairy cow research

**Methodology**	**Target genes**	**Milk characterization**	**Ref**
Milk composition			
GC–MS and LC–MS	Hippurate and ribose 5-phosphate	Organic whole milk	[43]
NMR spectroscopy	Choline, N-acetyl hexosamines, creatinine, glycerophosphocholine, glutamate, glucose 1-phosphate, galactose 1-phosphate, and orotate	Total protein content	[44]
NMR spectroscopy; GC–MS	Lactate, acetate, glutamate, creatinine, choline, carnitine, galactose 1-phosphate, and glycerophosphocholine, uracil, and lactic acid	The coagulation conditions of milk and milk traits	[45]
Milk quality			
GC–MS analysis	Glycine, Valine, malic acid, talose, hydroxyglutaric acid, fructose, and glucose	Goat milk, cow milk, pasteurized milk, ultra-high temperature-treated milk or cow milk	[46]
NMR spectroscopy analysis	Succinic acid and choline	Cow milk	[47]

*GC–MS* Gas chromatography-mass spectrometer, *LC–MS* Liquid chromatography-mass spectrometer, *NMR* Nuclear magnetic resonance

## Application of omics technology in ruminants

Animal farming is crucial to supplying local populations with high-quality protein in tropical nations. Animal production will confront two significant obstacles in the upcoming years: an expanding human population and a growing demand to cut greenhouse gas emissions. As a result, there will be a greater demand for animal products and a need to limit the emissions that ruminant agriculture, particularly beef and dairy intensive production, is responsible for, such as methane [48]. In tropical areas, there will likely be a comparable rise in consumer demand for sustainable practices and animal products. Better management and production methods are required to meet the need to enhance animal production output [49].

In the context of animal agriculture, novel omics technologies are being used more and more frequently. Animal production has made use of genetics, nutrigenomics, transcriptomics, proteomics, and metabolomics. The study of animal metabolism in response to a specific stimulus is made possible by the application of these omics’ technologies. This makes it possible to examine the molecular effects of environmental factors (including temperature and humidity), nutrition, gender, and welfare on the physiology of farm animals. The combination of these technologies also makes it possible to thoroughly examine the metabolism of animals, tissues or cells from genotype to phenotype [50]. Furthermore, by identifying the proteins responsible for desired characteristics like milk protein composition and meat tenderness using proteomics, the quality of dairy and meat products may be assessed [51]. In recent years, a number of omics applications in animal science have been thoroughly studied [51–53]. This review attempts to describe topics significant for tropical and subtropical animal production that were investigated using omics technology.

Nutrigenomics is the study of "the genome-wide influences of nutrition" [54] and how this "affects the balance between health and disease by altering the expression and/or structure of an individual’s genetic makeup" [55]. This definition emphasizes the fundamental idea that dietary nutrients modify gene expression, either directly or indirectly [56], impacting cell metabolism and/or signaling, protein expression, and ultimately, the health of tissues, organs, and the body as a whole. The idea that dietary components influence biological processes by interacting with cells' molecular environments has completely changed the way that nutrition is studied [57]. Additionally, the discipline of nutrition has started to take advantage of the technology and accompanying analytical tools introduced in the post-genomic period in order to get around these problems and investigate the association between genes and food more thoroughly. Nutrigenomics and nutrigenetics, two fields that use different methods to explain how genes and diet interact, but share the ultimate goal of optimizing health through diet personalization, have emerged as potent tools for figuring out the intricate relationships between genetic polymorphisms, nutritional molecules, and the biological system as a whole. The main reason for the reluctance to embrace these new fields is the concern that producing an excessive amount of biological data in a single study will drown the original question [54–57]. Nevertheless, the purpose of this review is to present nutrigenomics and nutrigenetics as the new faces of nutrition that, when paired with more traditional methods, will offer the essential building blocks to reach the lofty objective of nutritional intervention-based health optimization. In this situation, nutrients serve as more than just a source of energy and "building blocks" for cells; instead, they are bioactive molecules that are recognized by cellular sensors and cause a change in the biology of the cells [56, 57]. However, dietary elements that are not specifically bioactive, such as dietary calorie restriction, can nonetheless have nutrigenomic effects [58]. It is now possible to precisely tailor an organism’s biology by dietary manipulation since nutritional substances can interact with the DNA, notably through transcription regulators. Dietary substances interact with transcription factors to either directly or indirectly alter gene expression. Among these, ligand-dependent nuclear receptors, particularly peroxisome proliferator-activated receptors (PPAR) and liver X-receptor, are the most important for nutrigenomics [59].

## Application of omics technology in rumen microbiota

The core community of the complex anaerobic rumen microbial ecology, which is populated by bacteria, protozoa, fungus, methanogenic archaea, and bacteriophages, is made up of poorly described microbes [60]. However, it is known that the rumen microbiota is adept at fermenting complex fibrous substrates into compounds that, along with the microbial biomass, are used by ruminants to support their upkeep, development, and lactation [61, 62]. Tropics-based emerging nations have regions with a rich variety of plants. Ruminants may be crucial in converting those cellulosic plant components into beneficial sources of animal protein for human consumption in such regions [63, 64]. However, in tropical areas, most ruminants are fed on agricultural crop wastes and agro-industrial by-products that frequently contain high levels of lignocellulosic materials and low levels of good-quality protein. These feeds also typically contain low amounts of low-quality forages. In addition, lengthy dry seasons with high temperatures that are typical, and a lack of sufficient feed are the key factors that limit animal productivity. Therefore, one of the most crucial research priorities for ruminant production in the tropics and subtropics is the manipulation of the ruminal microbiome to enhance the utilization of tropical forages and maximize ruminant productivity while minimizing harmful environmental outputs like methane emissions [22, 65].

It has been frequently utilized to characterize the rumen microbiota by sequencing the 16S ribosomal RNA gene’s target areas. The rumen microbial population is described using this method; their function is not described. However, the development of bioinformatics tools and omics technologies, such as metagenomics, transcriptomics, metaproteomic, and metabolomics, allows for a deeper understanding of the rumen microbial ecology, particularly with regard to the relationship between the host and the microbe and the effect of dietary approaches on the performance of the animal [62]. While meta-transcriptomics can shed light on the actual function of microbiomes through gene expression, metagenomics enables evaluation of both the variety and prospective functional capacity of the microbiome [66]. For gaining access to the proteins expressed and the metabolites produced by NMR- or MS-based approaches, they also provide information on variously expressed metabolic pathways. Animals from tropical regions' rumen microbiomes are currently being studied using functional metagenomics and next-generation sequencing techniques [67].

Since low-quality roughages make up the majority of the diet of ruminants in many tropical regions, as opposed to temperate ones [68, 69], it is crucial to know which enzymes cause the dietary fiber to be degraded and which major microorganisms are responsible for its synthesis of fiber, particularly roughages through metabolomic technique [70, 71]. Omics technologies have been used in numerous research areas to provide light on the breakdown of fibrous carbohydrates and how diet affects rumen methane output [70, 72]. It is also becoming more common to use metabolomics to investigate the microbial activity in the rumen, and this application’s significance is related to the fact that it can offer crucial biochemical insights into the function of host-diet interactions in the rumen. In fact, the rumen produces a number of metabolites during the microbial fermentation and digestion of feedstuffs, including volatile fatty acids, organic acids, sugars, amino acids, and amines. These metabolites are then employed by the bacteria for growth before being absorbed and utilized for the production of meat and milk as well as for bodily upkeep [73].

## Applications of omics technology in milk production

Milk and dairy products are providers of the vital nutrients needed for development and growth. Lactose, fat, protein, minerals, and water comprise the main components of milk. Depending on the species, the individual, the stage of lactation, and other environmental circumstances, milk content varies. In addition, milk is a source of important nutrients for humans, representing an essential contribution to supply energy, protein, calcium, essential fatty acids (FAs), vitamins and bioactive compounds [74]. Omics is a method to evaluate the effects and mechanisms of feed supplements and use nutrient interventions to improve milk contents and develop innovative feed resources and management protocols. In the meanwhile, those techniques support dairy farmers in modifying the dietary composition and improving management to boost production effectiveness and avoid transitional diseases. Additionally, they offer viable remedies for reducing greenhouse gas emissions. Omics analyses have previously demonstrated their advantages in detecting molecular markers and predicting functional chemicals, but it is currently unable to offer an immediate response as a monitoring approach due to sampling time points, high cost, and present technology limitations. With enhancement, multi-omics could become a useful tool for assessing feeding and management, as well as monitoring the health of animals [75].

### Milk proteomics

Using the application of high-resolution 2-dimensional gel electrophoresis (2-DE), mono- and multi-dimensional liquid chromatography (LC), or MS, proteomics is a potent tool that can simultaneously evaluate numerous proteins in complicated combinations [76]. Proteomics has been applied to milk since the beginning of the 1990s. The detection and identification of new proteins, particularly minor proteins or proteins found in subcellular compartments including the milk fat globule membrane (MFGM-type 2) variations in the protein profiles depending on the species of mammal or the stage of lactation posttranslational modifications (glycosylation, phosphorylation, and other modifications), which occur at an incredibly high frequency naturally have all observed significant advancements. Recently, two extremely thorough evaluations discussed all of these advancements [38]. Nevertheless, over the past five years, a significant increase in the discovery of minor milk proteins has been indicated. These discoveries will aid in the characterization of the pathways and mechanisms that occur during lactation and provide details of the biological activity and functionality of these essential proteins [77]. The studies performing protein analysis of milk using LC–MS are show in Table 4.

**Table 4:** Studies performing protein analysis of milk using LC–MS

**Characteristics of milk protein**	**Analytical technique**	**Animal species**	**Ref**
All proteins	RP-HPLC	Cow	[78]
MFGM	Micro-capillary-HPLC	Cow	[38]
Caseins, whey	RP-HPLC	Cow	[79]
MFGM	Nano-LC	Sheep	[80]

*MFGM* Milk fat globule membrane, *RP-HPLC* Reversed-phase high performance liquid chromatography, *LC* Liquid chromatography

### Milk lipidomics

Lipids are one of the major components of milk and are present at 3%–5% in milk of most farm species. With an estimated fatty acid (FA) class of over 400, the theoretical number of triglyceride (TAG), phospholipid and sphingolipid molecular class present in milk is very large. The major lipids in cow’s milk are triacylglycerols, and the milk fat secretion is visible as little drops, comprising 97%–98% of the total lipid, diacylglycerols, monoacylglycerols, phospholipids, free fatty acids, and cholesterol and its esters constitute to the remaining lipids [81].

A potential technique for finding biomarkers for mastitis and heat stress in dairy cows is milk lipidomics [82]. The 56 screened lipid classes were divided into the following categories: two as cardiolipin (CL), four as bis-methyl phosphatidic acid (BisMePA), two as diacylglycerol (DG), one as hexosylceramide (Hex1Cer), six as Hex2Cer, nine as phosphatidylcholine (PC), eight as phosphatidylethanolamine (PE), two as phosphatidylserine (PS), three as sphingomyelin (SM), and nineteen as triacylglycerol (TG). In general, in this study conducted citrus peel extract as a material that contains bioactive compounds, such as flavanones (e.g. naringin and hesperidin) and o-polymethoxylated flavones (e.g. nobiletin and tangeretin), while the control group of supplemented citrus peel extract (at 0 g/d/cows) exhibited higher levels in milk of 42 lipid species from the groups CL, BisMePA, DG, Hex1Cer, Hex2Cer, PC, PE, PS, SM, and TG. However, the milk of control cows contained lower concentrations of 16 SM and TG-related lipid class [83].

Ultra-high performance liquid chromatography-quadrupole time-of-flight mass spectrometry (UHPLC–Q-TOF-MS) was used to identify 335 lipid class in cow milk, including 114 TGs, 15 DGs, 9 CLs, 7 phosphatidic acids (PAs), 70 PCs, 7 PEs, 20 PGs, 6 PSs, 8 Cers, 22 SMs, 6 HexCers, and 18 Hex2Cers. A further investigation used LC–MS to identify 3,454 TG molecules in cow milk that belonged to 220 TG categories (36 saturated TGs, 37 monounsaturated TGs, 37 di-unsaturated TGs, and 110 polyunsaturated TGs) [83]. In Ultra-high temperature (UHT) and reconstituted milk (whole milk), 577 lipid molecules, including 312 TGs, 16 DGs, 2 monoglycerides (MGs), 43 PCs, 65 PEs, 8 phosphatidylglycerols (PGs), 9 phosphatidylinositols (PIs), 2 PSs, 9 lysophosphatidylcholines (LPCs), 6 lysophosphatylethanolamines (LPEs), 12 CLs, 14 ceramides (Cers), 6 glycosylceramides, 12 diacylglycerophosphoethanolamine glycans, 26 sphingomyelins (SMs), 3 wax esters (WEs), 1 stearamide, and sphingosine (So) were identified using UPLC–Q-Exactive Orbitrap-MS [84]. Applying UPLC–Q-Exactive Orbitrap-MS, a total of 756 lipid molecules from 14 lipid subclasses (including 2 acyl carnitines (AcCas), 5 CLs, 45 Cers, 17 LPCs, 4 LPEs, 36 PCs, 80 PEs, 9 PGs, 22 PIs, 55 HexCers, 56 SMs, 17 FAs, 15 DGs, and 416 TGs) [82]. Table 5 shows lipidomic application in ruminant milk fat products.

**Table 5:** List of lipidomic application in milk fat products and nutrient of milk synthesis stage based on ruminants

**Characteristics of Lipid**	**Analytical technique**	**Animal species**	**Ref**
SFA, MUFA, PUFA and CLA	GC and LC–MS/MS	Cow	[83]
35 lipids, 9 CLs, 7 PAs, 70 PCs, 7 PEs, 20 PGs, 6 PSs, 8 Cers, 22 SMs, 6 HexCers, 18 Hex2Cers, 15 DGs, 114 TGs	UHPLC–Q-TOF-MS	Cow	[84]
97.75% TG, 1.81% Chol + DG + FFA, 0.04% CE, 0.04% MG, 5.10% LacCer, 36.58% PE, 6.18% PI, 7.28% PS, 24.60% PC, 20.25% SM	HPLC-ELSD	Cow	[85]
103 lipids, 17 PCs, 15 SMs, 11 Cers, 3 HexCers, 25 TGs, 32 DGs	UPLC–Q-TOF-MS	Goat	[86]
756 lipids in 14 lipid subclasses, 5 CLs, 45 Cers, 17 LPCs, 4 LPEs, 36 PCs, 80 PEs, 9 PGs, 22 PIs, 2 AcCas, 55 HexCers, 56 SMs, 17 FAs, 15 DGs, 416 TGs	UPLC–Q-Exactive Orbitrap-MS	Goat	[82]
97.32% TG, 1.89% Chol + DG + FFAs, 0.04% CE, 0.10% MG, 7.57% LacCer, 29.17% PE, 5.77% PI, 7.65% PS, 26.25% PC, 23.24% SM	HPLC-ELSD	Goat	[85]

*SFA* Saturated fatty acids, *MUFA* Monounsaturated fatty acids, *PUFA* Polyunsaturated fatty acids, *CLA* Conjugated linoleic acid, *CLs* Cardiolipin, *BisMePA* Bis-methyl phosphatidic acid, *DG* Diacylglycerol, *Hex1Cer* Hexosylceramide, *PC* Phosphatidylcholine, *PE* Phosphatidylethanolamine, *PS* Phosphatidylserine, *SM* Sphingomyelin, *TG* Triacylglycerol, *PA* Phosphatidic acid, *PG* Phosphatidylglycerol, *PI* Phosphatidylinositol, *LPC* lysophosphatidylcholine, *LPE* lysophosphatylethanolamine, *MG* Monoglyceride, *Cer* Ceramide, *SM* Sphingomyelin, *AcCa* Acyl carnitine, *LacCe*r Lactosylceramide, *Chol* Cholesterol, *FA* fatty acid, *FFA* Free fatty acid, *LC–MS* Liquid chromatography-mass spectrometer, *GC* Gas chromatography, *UHPLC–Q-TOF-MS* Ultra-high performance liquid chromatography-quadrupole time-of-flight mass spectrometry, *HPLC-ELSD* High performance liquid chromatography evaporative light scattering detector

### Milk glycomics

Mammalian milk contains large amounts of free oligosaccharides, which perform crucial roles in the development of the immune system, prebiotic chemicals, and the immune system’s defense. Nano flow liquid chromatography was used to measure milk oligosaccharides on chip-based devices. Additionally, it was used to discover what proportions of fucosylated and sialylated milk oligosaccharides were present in primates. Cluster analysis of monkey milk was carried out utilizing the chromatographic profiling for a systematic and thorough investigation of the evolutionary patterns of milk oligosaccharides [87]. The oligosaccharide content of milk from other mammalian species was thoroughly examined in the last years to determine the best sources of nutrients and bioactive substances for newborn milk formula. The studies performing oligosaccharide analysis of milk using LC–MS are show in Table 6.

**Table 6:** Summary of studies performing oligosaccharide analysis of milk using LC–MS

**Animal species and product**	**Stages and composition of oligosaccharide in milk**	**Analytical technique**	**Ref**
Dairy cows’ colostrum	First lactation period	HPLC-Chip	[88]
Dairy cows’ colostrum and milk, infant formula	Detection of six different oligosaccharides	HILIC	[89]
Dairy cows’ colostrum	Structural assignment	HILIC	[90]
Goat	Comparative analysis in different goat breeds	UPLC	[91]

*LC–MS* Liquid chromatography-mass spectrometer, *HPLC* High performance liquid chromatography, *HILIC* Hydrophilic interaction chromatography, *UPLC* Ultra performance liquid chromatography

### Milk vitamins

Vitamin content is evaluated in the majority of studies on other milk components, with vitamin D receiving the greatest attention. The two forms of vitamin D, vitamin D_2_ (ergocalciferol) and vitamin D_3_ (cholecalciferol), both have a steroidal structure. The primary techniques for detecting vitamin D nowadays are based on reversed-phase HPLC, typically using a C_18_-packed column and mass spectrometry or UV detection as shown in Table 7 [82]. The amounts of vitamin D_3_ in fresh cow milk, commercial and fortified milk, and a dairy-based infant formula were determined using reliable methods employing LC–MS and LC–tandem MS. The authors authenticated the vitamin D levels in each milk sample without the need of extensive sample pre-treatment. Using LC–MS, a comparable strategy was adopted to evaluate the levels of vitamin D in cows' milk, milk powder, and newborn formula [92].

**Table 7:** Summary of studies on milk metabolomics for milk authenticity and analysis of other milk constituents using LC–MS

**Metabolite**	**Analytical technique**	**Animal species and sample**	**Ref**
Vitamin D_3_	microchip LC	Cow fresh milk, commercial milk, fortified milk, infant formula	[92]
Vitamin D_3_	UPLC	Cow milk, infant formula	[82]
Melatonin	RP-HPLC	Cow milk	[93]
Metaboles	UHPLC	Cow milk	[94]
Metabolome	UPLC	Cow milk	[95]

*LC–MS* Liquid chromatography-mass spectrometer, *RP*-*HPLC* Reversed-phase high performance liquid chromatography, *UHPLC* Ultra high-performance liquid chromatography, *UPLC* Ultra performance liquid chromatography

## Using omics technologies to measure meat quality

Animal products, especially meat, are a good source of bioactive compounds, including vitamins, minerals, peptides, and fatty acids, that have beneficial effects on human health. New, healthier meat products must be developed in the face of increased worldwide rivalry among meat producers and rising food consumer awareness. Producers employ dietary supplements with functional characteristics for animal meals and as direct additions to meat products to achieve these standards. Each one is examined in terms of how it affects human health as well as some of the final products' high-quality characteristics [96]. Growth promoters have been widely used by animal producers in the meat production chain for many of years to enhance not only growth performance but also the qualities of the carcass and the meat, though the effect on these qualities continues to be up for discussion [97]. Hence, this review enhances our understanding of how the omics methods are effective in examining the underlying differences in meat quality caused by different genes, proteins, and metabolites. The biomolecular signs that have been found can be used as biomarkers for predicting quality features and provide light on molecular interactions. Recent research has effectively investigated the effects of several physiological systems on meat quality during meat production using these technologies.

Several different aspects of meat quality determine whether a particular meat cut is appropriate for human consumption [98]. The use of omics as instruments to describe or regulate the quality of diets for muscles was proven by Gagaoua [99]. In several published papers, methods such as transcriptomics, targeted and untargeted proteomics, metabolomics, and genomics were used to characterize the safety, adulteration, and authenticity of meat and meat products as well as to evaluate meat quality and to determine the molecular profiles of meat and milk products [75]. Munekata et al. [100] reported that the foodomics is a valuable tool to understand biochemistry and relevant compounds associated with meat quality traits. This approach is composed of four main strategies (transcriptomics, metabolomics, proteomics and lipidomics) that allow comprehensive and high-throughput characterization of the genetic expression, metabolites, proteins and lipids of meat and meat products to improve the knowledge about the underlying mechanisms and molecules involved in the meat quality traits to produce high-quality cuts [101].

Chanjula et al. [16] reported the impact of incorporating *Mitragyna speciosa* (Korth) Havil leaf powder into the diet of finishing goats on both carcass composition and meat quality. The methodologies outlined by Hara and Radin [102] for the extraction and methylation of fatty acids from meat were utilized. Following the process of extraction and methylation, the injection of each sample (1 mL) was carried out on a Finnigan GC Focus gas chromatograph. Fatty acids constitute the primary constituents of lipids, exerting a discernible influence on the meat’s overall quality. The quality of fatty acids is significantly influenced by their concentration, which in turn determines their saturation level. The fatty acid composition of biological tissues is affected by diet, as evidenced by a study conducted by Smith et al. [20], which found that goats fed pasture had a higher proportion of unsaturated intermuscular fat compared to those fed grain. The rapid solidification of long-chain saturated fatty acids (SFAs) has an adverse impact on the palatability of meat. In addition, the study conducted by Barido et al. [103] provided evidence indicating that the supplementation of methionine and lysine led to increased protein scores, improved water holding capacity, and decreased shear force scores. The supplementation of methionine, omega-3, beta-carotene, alpha-tocopherol, and conjugated linoleic acids have been found to have several effects on meat production. These effects include enhancing the redness of the meat, reducing lipid oxidation, improving meat tenderness, and increasing the protein content and percentage of oxymyoglobin. The water-holding capacity was assessed using the methodology outlined by the National Research Council (NRC) in 2000 [104], with minor adjustments made. The moisture content of both the raw material and the supernatant was assessed using the AOAC method as described by Kristensen and Purslow [105]. The determination of crude fat content was conducted using the Soxhlet system and the ether extraction method. The assessment of nitrogen content was performed using the Kjeltec system. The fatty acid composition was determined using an Agilent GC system equipped.

As previously stated, breed is a significant aspect that might affect the quality of the meat. This way, the characteristics of meat quality resulting from various breeds, muscles, rearing techniques, and post-slaughter processing circumstances might be explained by knowing how variations in gene expression affect the development of animals and metabolic processes [106]. The qualitative characteristics of meat (*Longissimus thoracis*; LT) from PDO database Maine-Anjou cows, for instance, were discovered to be influenced by rearing procedures and diet composition (hay, grass, and haylage), according to proteomics data [107]. On the right side of the animal’s carcass*,* LT muscle samples were eliminated, and four distinct components were identified. After that, the initial portion of the samples was placed in liquid nitrogen and stored at −80 °C until it was tested for fiber characterization (fiber area, myosin heavy chain isoforms (MyHC), and metabolic enzyme activities), as well as for protein extractions for Dot-Blot protein quantification. The protein extracts used in this study were determined according to the dye binding method of Bradford [108] using the Bio-Rad Protein assay. In another experiment, Gagaoua et al. [109] discovered that the structural proteins MyHC-I, MyHC-IIa, and MyHC-IIx, oxidative stress biomarkers DJ-1 and peroxiredoxin 6 (PRDX6), and proteolysis biomarkers such as Calpain-1 catalytic subunit (CAPN1) could all be utilized as biomarkers for tenderness. The quantification of the three different protein biomarkers associated with MyHC isoforms, which are classified as structural proteins, was conducted utilizing a suitable high-resolution mini-gel electrophoresis approach [109] (Table 8).

**Table 8:** List of omics technologies to measure meat quality

**Methodology**	**Sample Source**	**Target**	**Ref**
Finnigan GC Focus gas chromatograph	Finishing goats	Fatty acids profile	[16]
Soxhlet system	Hanwoo steers	Crude fat	[103]
Kjeltec system		Nitrogen content	
Agilent gas chromatography system		Fatty acid composition	
Adequate electrophoresis protocol	Beef	MyHC isoforms	[107]
Computerized image-analysis		Muscle fibers	
Bio-Rad Protein assay		Protein concentrations	

*GC* Gas chromatography, *MyHC* Myosin heavy chain

## Using omics technologies to mitigate methane emissions

Ruminants can consume feeds made of prokaryotic and eukaryotic microorganisms that break down cellulolytic and hemicellulolytic feedstuffs [110]. Hydrogen (H_2_), carbon dioxide (CO_2_), and formic acid are created as byproducts of fermentation throughout the breakdown process [111]. Methanogenic archaea in the rumen use H_2_ to degrade CO_2_ to create CH_4_, which raises greenhouse gas (GHG) levels [110, 111]. To reduce the amount of CH_4_ that ruminants create, a variety of mitigation techniques are being researched. These include changing food formulations [112] and using chemical inhibitors to lessen rumen methanogenesis [113]. The application of genetic and genomic selection to breed for lower CH_4_ production is anticipated to generate significant genetic advantages that are cumulative and permanent over generations, notwithstanding the efficacy of such methods [114]. Important insights into the role of rumen microbial structure and metabolite profiles have been gained through the use of metagenomic and metabolomic methods to understand the variance in microbial genomes and microbial profiles [115]. The assessment of the taxonomic and genetic makeup of complex microbial communities has advanced thanks to the availability of information on the relative abundance and identification of microbial species and genes in metagenomics. This has made it possible to identify and quantify microbial data to characterize the structure and functioning of microbial communities [116]. With the help of metabolomics, it is possible to identify a network of biological markers that reflect physiological and pathological events as they happen, highlighting phenotypic variations seen between extreme animal groups. The definition of multiple organic compounds existing in a biological tissue or fluid has been made possible by the application of metabolomics in ruminant research. Additionally, this evaluation of system wise metabolism and biology has advanced [53, 116].

## Conclusions and perspectives

This review provided an overview of recent studies on the functional of bioactive compounds, genomics, transcriptomics, proteomics, and metabolomics of meat and milk production in ruminants. An overview of omics technologies in nutritional research is presented, which focuses on recent applications of genomics, transcriptomics, proteomics, and metabolomics in functional and biological activities. In particular, meat and milk components were assessed using omics research based on the various methodologies of genomes, transcriptomics, proteomics, and metabolomics; omics studies reveal the response of the organs to factors; multi-omics studies connect them and demonstrate how the factors impact the body either directly or indirectly, as well as how the body reacts to the factor. Nevertheless, remain restrictions on single omics’ ability to combine various levels and organs and create an accurate representation of how specific elements affect meat and dairy products, including the methodology through which this impact is perceived. Multi-omics techniques enable researchers to screen additional genes associated with heritable traits, elucidating in ruminants’ physiology mechanisms as well as the pathology of metabolic illnesses. These potential techniques integrate all levels and organs to create an exhaustive overview of ruminant livestock production and generate novel directions of research for the field of ruminants. Guidelines for creating technologies that will provide access to more recent data is processomics which is a methodology that uses metabolomics to assess the shelf-life of commercially available shelf-stable goods. However, it faces challenges such as accurate identification and quantification of metabolites, diverse chemical composition, stability, sample complexity, large dataset size, and lack of standard reference NMR/MS spectra. Non-technical issues like limited availability of costly metabolome devices and lack of defined test techniques reduce comparability of metabolic fingerprints. Recent studies focus on analyzing volatile organic compounds (VOCs) using a metabolic fingerprinting technique based on mass spectrometry (MS). However, the analysis of the volatilome is influenced by various factors, including the source, composition, and microstructure of the food. Reliable calibration algorithms are needed to compare the practical impact of processing based on different types of chemical reactions. To improve precision, data standards, public processomics databases, and modular programming should be established. Using comprehensive processomics that combines analysis of VOC and nonVOC fingerprinting can uncover novel quality characteristics and unexpected ways in which quality is altered in response to external processing factors. Further analytical strategies should be implemented to extend chemical material covering and improve coverage.

## Data Availability

Not applicable.

## Abbreviations

AOAC: The Association of Official Analytical Chemists
CH_4_: Methane
CLA: Conjugated linoleic acid
CO_2_: Carbon dioxide
CT: Condensed tannins
DNA: Deoxy ribonucleic acid
FA: Fatty acid
GHG: Greenhouse gas
H_2_: Hydrogen
kDa: Kilodalton
LC: Liquid chromatography
LC–MS: Liquid chromatography-mass spectrometer
MFGM: Milk fat globule membrane
mRNA: Messenger ribonucleic acid
MS: Mass spectrometer
NMR: Nuclear magnetic resonance
PDO: PHP Data Object database
PNT: Phytonutrients
qRT-PCR: Quantitative reverse transcription polymerase chain reaction
RNA: Ribonucleic acid
SFAs: Saturated fatty acids
SP: Saponins
TAG: Triglyceride
UV: Ultra violet
VFAs: Volatile fatty acids
